# Letter from Dr. Isaac I. Greenwood, to the New York Editor

**Published:** 1841-09

**Authors:** Isaac I. Greenwood

**Affiliations:** 71 Warren-st.


### ARTICLE VIII.

*Letter from* DR. ISAAC I. GREENWOOD, *to the New York Editor.*

DEAR SIR: *New York, February* 27, 1841.

YOU will excuse the repeated liberties I have taken in addressing you, but seeing, under your signature, in the Journal of Dental Science, page 227, No. 9 and 10, vol. 1, a recommendation of the use of *arsenic* as a cure for the tooth-ache, I am urged, by a sense of duty, and by your endeavours to substantiate the facts of the usefulness of this mineral, when applied to that purpose, to give you my experience in the use of the same for six years past. I have, hitherto, been kept silent by a publication by H. Burdell, M. D., part 1, page 29, upon the "Diseases of the Teeth," wherein he deprecates the use of *arsenic* as a remedy for this evil, and gives the decided preference to *kreosote*. I have waited patiently, until some one more experienced in its virtues and uses as a cure for this evil, should speak its praise, and I am happy it has found its advocate in one I esteem so capable of judging of its curative or injurious effects as thus applied. The evil arising from the use of this mineral, is, in the "modus operandi," or mal-administration of it, as a remedy. The method I have used, I have ever found to produce the effects I desired, and I may say that every case I have had, since I have used it as a remedy, and I commenced with myself, has proved successful.

When a patient applies to me for the cure of tooth-ache, I examine the tooth, and clean out the cavity, endeavouring to make *bare* the nerve, if practicable, with a small instrument. If the nerve bleeds, so much the better. I then wipe out the cavity with raw cotton, steeped in essence of peppermint, laudanum, or alcohol. After which, I take raw cotton of sufficient size to stop up three-fourths of the cavity of the tooth. I dip the point into laudanum, so as somewhat to saturate the cotton with it, that the *mixture* I shall mention below may adhere to it. I then take up upon the point of it, by *touching the mixture*, about the size of a large *pin's head*, and in *no instance* do I ever *use more, however large the cavity in the tooth;* but sometimes a *smaller* quantity. This I place in the cavity of the tooth, immediately in contact, if

I can, with the *nerve,* and stop up the cavity with mastic, composed of Venice turpentine, heated, and mixed with calcined plaster of Paris and chalk. Feuchtwanger's Prussian cement for the teeth, *will answer,* placed upon the raw cotton in the tooth, and sometimes mixed up with it so as to fill up the cavity, charging the patient to take it out in *three* days *exactly,* and in no wise to masticate on that side during the time. If a patient will come to me, which they generally will do, I take it out for them, which I prefer to do, and wash out the cavity with alcohol. The tooth is by this time *cured ;* but for fear there may remain an ichorous fluid oozing still from the dental canal, I leave it for *three days longer,* when the organ is *fully prepared and ready* for stopping, either with *gold or otherwise.* The symptoms of the efficacy of the cure are these, viz: the pain, *after* commencing, will endure for three or four hours, sometimes more, according to the *irritability* of the patient. After the acute pains have passed away, a soreness will continue for some time, accompanied by a looseness of the organ, occasioned by the inflamed state of the periosteum. This gradually dies away, and by the second or third day, *in almost every case, disappears.* If, *when* the raw cotton and the mastic are removed on the third day, the patient takes *cold water* in the mouth, and *no pain* arises from it, the *cause* is removed. This is *the proof in all cases.* I have been thus prolix, in order that you may be supported by one who has *tested* its efficacy for years with success, and, indeed, I make use of no other remedy.

*Mixture.*—To be placed in *an ounce glass vial,* with *glass stopper.*

℞ Three parts arsenic,
One, do. acetate morphine,
Mix.

With sentiments of esteem, I am, &c.

ISAAC I. GREENWOOD, 71 *Warren-st.*

*Note.*—From the above description of the pain, which follows the application of arsenic and morphine, I should substitute kreosote in place of the morphine, inasmuch as the mixture which I have recommended in a former number, is found to give very little, if any uneasiness to the patient.—*New York Ed.*

